# The CXCL12-CXCR4 axis in colorectal cancer: immune regulation, metastatic progression, and therapeutic implications

**DOI:** 10.3389/fimmu.2026.1783222

**Published:** 2026-04-23

**Authors:** Shuanglin Tian, Jinrui Liu, Xiulan Yang, Jiawei Guo

**Affiliations:** 1 Department of Pharmacology, School of Medicine, Yangtze University, Jingzhou, China; 2 Second Clinical Medical College of Yangtze University, Jingzhou, China; 3 Department of Cardiology, The First Affiliated Hospital of Yangtze University, Jingzhou, China

**Keywords:** chemokine, colorectal cancer, CXCL12, CXCR4, immunotherapy, metastasis, tumor microenvironment

## Abstract

Metastasis remains the major cause of mortality in colorectal cancer (CRC) despite continued advances in diagnosis and treatment. Increasing evidence identifies the CXCL12/CXCR4 chemokine axis as a critical driver of CRC progression and metastatic dissemination. Through dynamic interactions between tumor cells and the tumor microenvironment, this axis regulates multiple processes essential for metastasis, including driving migration and invasion, angiogenesis and lymphangiogenesis, and shaping the tumor immune microenvironment through recruitment of immunosuppressive populations, blockade of effector lymphocyte trafficking and function, and modulation of immunosuppressive cytokines including IL−10. In this review, we summarize the molecular mechanisms by which CXCL12/CXCR4 promotes CRC metastasis. These pleiotropic effects are mediated by crosstalk with PI3K/Akt, MAPK/ERK, and Wnt/β−catenin pathways, and are regulated at transcriptional, post−transcriptional, and post−translational levels. Preclinical studies demonstrate that CXCR4 antagonists (e.g., plerixafor, LY2510924) suppress metastasis and, when combined with immune checkpoint inhibitors, can reverse the “cold” immune phenotype of microsatellite−stable CRC. We also discuss recent advances in the regulation of CXCL12/CXCR4 expression, the role of related receptors such as CXCR7, and emerging strategies targeting this axis for therapeutic intervention. Collectively, current evidence supports the CXCL12/CXCR4 axis as a promising biomarker and therapeutic target in metastatic CRC, and further elucidation of its regulatory network may facilitate the development of more effective precision treatment strategies.

## 1 Introduction

Colorectal cancer (CRC) is one of the malignancies with the highest incidence and mortality rates worldwide. According to GLOBOCAN 2022 statistics, there were over 1.93 million new CRC cases and more than 904,000 deaths globally, ranking third in incidence and second in mortality among all malignancies (1). The clinical course of CRC exhibits the characteristic “adenoma-carcinoma sequence” progression. Despite advances in surgical and targeted therapies that have improved treatment outcomes, the 5-year survival rate for CRC patients with distant metastasis remains extremely low, with over 50% of patients ultimately dying from tumor metastasis (2). CRC metastasis is a complex process involving multi-step, multi-factor regulation, and its precise molecular mechanisms have not yet been fully elucidated. Therefore, in-depth investigation of the regulatory network of CRC metastasis and the search for effective intervention targets represent an urgent task in current research.The role of chemokines and their receptors in tumor progression has garnered increasing attention in recent years (3–6).

The human chemokine family comprises approximately 50 members that primarily regulate immune cell migration and function through G protein-coupled receptors (7). Among these, the signaling axis formed by CXCL12 (stromal cell-derived factor-1, SDF-1) and its receptors CXCR4 and CXCR7 occupies a unique biological position. Unlike most family members that exhibit ligand-receptor redundancy, CXCL12 is the only homeostatic chemokine that specifically binds CXCR4 without alternative chemokine ligands. Notably, CXCL12 possesses dual functions of homeostatic and inflammatory chemotaxis: maintaining tissue homeostasis under physiological conditions while being upregulated and participating in pathological processes in inflammatory or tumor microenvironments. The binding of CXCL12 to its receptors is highly specific-knockout of either the CXCL12 or CXCR4 gene leads to perinatal death in mice due to developmental defects in the heart, nervous system, and hematopoietic system. This lethal phenotype profoundly reveals the central role of this axis in embryonic development and organ homeostasis, with functions extending far beyond immune regulation (8–11).

Based on this homeostatic characteristic, the CXCL12/CXCR4 axis provides a crucial theoretical foundation for understanding organ-specific tumor metastasis. At the beginning of this century, Zlotnik and colleagues pioneeringly proposed the “organ-specific metastasis” hypothesis: tumor cells express chemokine receptors (such as CXCR4) and respond to chemokine ligands (such as CXCL12) secreted by stromal cells in specific organs (e.g., lung, liver, lymph nodes), thereby directionally migrating to these “soil” to form metastatic lesions (12, 13). This discovery opened a new era in tumor chemokine research and established the CXCL12/CXCR4 axis as a key regulator of organ-specific tumor metastasis. Subsequent studies further confirmed that beyond CXCR4, CXCL12 also binds with high affinity to another receptor-CXCR7 (also known as atypical chemokine receptor 3, ACKR3) (14–16). Unlike the classical G protein-coupled receptor CXCR4, CXCR7 belongs to the atypical chemokine receptor family, with unique structural features in its intracellular region. It primarily signals through β-arrestin recruitment to activate downstream pathways (such as ERK and Akt), while also functioning as a scavenger receptor that internalizes and degrades CXCL12 to regulate extracellular ligand concentrations, thereby indirectly modulating CXCR4-mediated signal transduction (17). Furthermore, CXCR7 can form heterodimers with CXCR4, enhancing or interfering with CXCR4’s chemotactic function and creating a more complex signaling network (14, 15).

Of note, the ligand recognition mechanism of CXCR4 exhibits complexity and diversity.In addition to the classical ligand CXCL12, other chemokines and atypical ligands can participate in CXCR4 signaling regulation (18). For instance, CXCL14, although not directly binding to CXCR4, can form heteromeric complexes with CXCL12 to enhance CXCL12-induced chemotactic activity [10]; macrophage migration inhibitory factor (MIF) has been confirmed to bind CXCR4 as an atypical ligand, activating downstream signaling pathways and promoting tumor cell survival (19). high mobility group box 1 (HMGB1) can also form complexes with CXCL12 to mediate inflammation and pre-metastatic niche formation in a CXCR4-dependent manner (20). In these cases, altered ligand binding patterns can lead to biased activation of downstream signaling pathways, such as switching from classical G protein signaling to β-arrestin recruitment, thereby affecting cell migration, survival, and drug resistance phenotypes. Moreover, CXCR4 signal output is finely regulated by receptor heterodimerization. CXCR4 not only forms heterodimers with CXCR7, modulating ligand binding affinity and signal transduction direction, but also interacts with other chemokine receptors such as CCR2. The formation of CXCR4-CCR2 heterodimers can alter receptor phosphorylation patterns, internalization dynamics, and the activation intensity of downstream MAPK pathways, producing functional synergy or antagonism in the interaction between tumor cells and the microenvironment (21).

CRC, as a highly heterogeneous gastrointestinal malignancy, has a complex genetic basis involving cumulative mutations in multiple driver genes such as APC(Adenomatous Polyposis Coli), KRAS(Kirsten Rat Sarcoma Viral Oncogene Homolog), and TP53(Tumor Protein P53), often accompanied by genetic syndrome backgrounds including Lynch syndrome and familial adenomatous polyposis (22). In this process, the continuous interaction between tumor cells and their microenvironment is the key driving force for tumor invasion and metastasis. Cancer-associated fibroblasts (CAFs), tumor-associated macrophages (TAMs), regulatory T cells (Tregs), and endothelial cells in the tumor microenvironment secrete various chemokines, growth factors, and extracellular matrix (ECM) remodeling enzymes, synergistically promoting malignant progression (23). In this context, the CXCL12/CXCR4/CXCR7 axis plays a particularly important role in CRC, with its mechanism involving not only the migratory capacity of tumor cells themselves but also the recruitment and functional reprogramming of multiple cell types in the tumor microenvironment (24).

Studies have shown that CXCR4 and CXCR7 are significantly overexpressed in CRC cell lines and primary tumor tissues, with differential expression levels among tumor cells, normal epithelial cells, immune cells, and stromal cells (24, 25). The positive expression rate of CXCR4 in cancer tissues was 100% (16/16).The positive expression rate of CXCR4 in liver metastases was 100% (5/5) (26). Only 50% of the normal mucosa expresses it (in more than 10% of the cells), and the expression level is significantly lower than that in cancer tissues. The CXCR4 positive rate in the SW480, SW48 and SW620 cell lines is as high as 60-80% (26). Approximately 25% of CRC cases exhibit CXCR4 expression in tumor-associated microvascular endothelial cells, which is significantly correlated with TNM stage, lymph node metastasis, and distant metastasis, suggesting its involvement in tumor angiogenesis (27). This discrepancy may arise from differences in tumor stage, stromal composition, or detection methods, highlighting the context-dependent nature of CXCL12 expression.: some studies report a 2–3 fold downregulation of CXCL12 mRNA in tumor tissues compared to normal tissues (28), while others report its upregulation (29). At the cellular level, CXCL12 is primarily secreted by stromal cells such as CAFs, with tumor cells themselves showing low expression, forming a “receptor high-expression, ligand low-expression” pattern that enables tumor cells to respond to exogenous CXCL12 signals for directional migration (30). CXCR7 is upregulated in both CRC tumor cells and tumor-infiltrating immune cells (such as tumor-associated macrophages, TAMs), with expression levels approximately 3–10 fold higher than in normal epithelial cells (24). Its distribution involves both cytoplasmic and perinuclear regions, suggesting participation in ligand transport and signaling regulation. From normal mucosa → adenoma → adenocarcinoma → liver metastasis, CXCR4 expression shows a progressive increasing trend, with CXCR4 mRNA levels in metastatic lesions more than 2-fold higher than in primary lesions (31). while CXCL12 is downregulated in primary lesions, it can be re-induced and upregulated in residual tumors after radiotherapy or chemotherapy, suggesting that therapeutic stress can reshape the chemokine microenvironment (29).

CXCR4 overexpression has been confirmed by the latest meta-analysis to be closely associated with significantly shortened overall survival (pooled HR = 2.70) and disease-free survival (pooled HR = 2.68) in CRC patients, serving as an independent poor prognostic factor (32). Similarly, high CXCL12 expression is significantly associated with lymphovascular invasion, lymph node metastasis, and worse 5-year overall survival rates (3). Notably, although CXCR4 and CXCR7 share the same ligand, they exhibit different signaling regulation and functional effects in CRC cells: gene expression profiling analysis shows that CXCR4- and CXCR7-overexpressing cells display completely different gene regulation patterns upon CXCL12 stimulation, with some genes even showing opposite expression (33). Functionally, CXCR7 overexpression is closely related to chemotherapy resistance in CRC cells, mediating resistance to oxaliplatin through regulation of tumor cell metabolic activity (34).

Importantly, the combined marker consisting of CXCR4, CXCR7, and CXCL12 demonstrates higher predictive efficacy: in clinical cohorts, it predicts metastasis with 86.67% sensitivity and 97.06% specificity (OR = 2.72, p=0.0014), and in the TCGA database, the OR value for predicting overall survival reaches 4.04 (p=0.0006), establishing it as an independent molecular marker for predicting metastasis risk and poor prognosis in CRC patients (35).

In conclusion, the CXCL12/CXCR4 axis plays a core driving role in the CRC metastatic cascade. An in-depth understanding of its expression regulation, signal transduction, and interaction mechanisms with the tumor microenvironment—including its crosstalk with other receptors such as CXCR7—holds significant theoretical value and clinical implications. This review systematically summarizes the role and molecular mechanisms of this axis in CRC metastasis and explores its potential as a clinical therapeutic target, with the aim of providing a scientific basis for improving treatment outcomes and prognosis for CRC patients.

## 2 The role of the CXCL12/CXCR4 axis in CRC metastasis

The CXCL12/CXCR4 axis functions as a master chemoattractant, orchestrating the precise migration of CXCR4-expressing cells along CXCL12 concentration gradients (36, 37). This mechanism, while indispensable for physiological processes such as hematopoiesis (38) and immune cell trafficking, is frequently hijacked during malignant progression to drive tumor dissemination. CRC exemplifies the clinical consequences of this pathological appropriation. As the third most common malignancy worldwide and the second leading cause of cancer-related mortality, CRC claims nearly one million lives annually, with metastatic disease accounting for the overwhelming majority of deaths (39, 40). Consistent with this, elevated CXCR4 expression in primary CRC strongly correlates with advanced TNM stage, distant metastasis and diminished overall survival (41).

CRC dissemination proceeds through a highly coordinated, multistep cascade in which CXCL12 acts as a central conductor (42). Within the tumor microenvironment, CXCL12 secreted by CAFs (43, 44), endothelial cells (45) and tumor cells (42) themselves directs disease progression through distinct yet interconnected spatiotemporal phases. We first examine how this signaling axis governs tumor cell-autonomous migration, epithelial–mesenchymal transition and ECM remodeling—critical events that enable tumor cells to extravasate from the primary site and intravasate into metastatic sites (42, 44). We then consider its essential role in orchestrating angiogenesis and lymphangiogenesis, which supply oxygen and nutrients to the tumor and provide the anatomical conduits for systemic spread (46, 47). Finally, we explore how CXCL12-CXCR4 signaling extensively reprograms the immune tumor microenvironment, establishing broad immunomodulatory networks that actively subvert anti-tumor immunity (48, 49). A mechanistic understanding of these interconnected phases is essential for the rational design of targeted therapeutic strategies against metastatic CRC.

### 2.1 Driving migration and invasion

Metastatic dissemination of CRC requires coordinated changes in tumor cell behavior, including enhanced motility, phenotypic plasticity, and the ability to breach extracellular barriers. The CXCL12/CXCR4 axis is a major regulator of these processes and plays a central role in CRC migration, invasion, and organ-specific metastasis.

Among these mechanisms, chemotactic guidance is an important early step. CXCL12 gradients in the primary tumor microenvironment and distant organs provide directional signals for CXCR4-expressing CRC cells (36, 37). This chemotactic effect is particularly relevant to the preferential metastasis of CRC to the liver (50) and lungs (42), where CXCL12 is abundantly expressed. Upon ligand binding, CXCR4 activates downstream pathways such as PI3K/Akt, MAPK/ERK, and FAK/Src, which promote cell polarization, cytoskeletal reorganization, and directional motility (42, 51, 52). These signaling events allow CRC cells not merely to respond passively to chemokine gradients, but to actively migrate toward permissive metastatic sites. Collectively, these findings support the notion that CXCL12/CXCR4-directed chemotaxis represents a fundamental early step in organotropic CRC metastasis.

Tumor cells must adhere to the basement membrane and secrete proteases to degrade it and the ECM during metastasis,thereby gaining access to the surrounding stroma (53). The interaction between CXCR4 and CXCL12 critically modulates the activity of cell adhesion-related factors such as MMPs and integrins, thereby facilitating the metastasis of CRC cells (51, 54).

Tumor cells secrete MMPs that degrade adhesion proteins, including basal membrane collagen and interstitial collagen, thereby destroying the basement membrane and facilitating tumor metastasis and invasion (55). CXCL12/CXCR4 upregulates MMP-2/9 via PI3K/Akt and MAPK/ERK-Ets-1signaling, degrading type IV collagen to facilitate CRC cell invasion (42, 56). This dual pathway ensures robust matrix metalloproteinase (MMP) expression, as CXCR4 knockdown or AMD3100 (plerixafor, a CXCR4 antagonist that blocks CXCL12 binding) treatment abrogates MMP-2 and MMP-9 activity in CRC cells (54, 57).

Integrins, a family of cellular adhesion molecules, are highly expressed in tumor cells, where they facilitate connections between the ECM and intracellular skeleton proteins, and acting as a receptor to bind extracellular signaling molecules, thereby influencing MMP expression in tumor cells (58). Beyond this role, integrins enhance tumor cell migration by activating focal adhesion kinase (FAK) and SRC family kinases (SFKs) (52). The CXCL12/CXCR4 axis upregulates integrin αvβ6 through ERK/Ets-1 pathway activation, promoting directional migration of CRC cells (51). Integrin αvβ6 expressed by CRC cells can activate TGF-β in fibroblasts, stimulating further CXCL12 secretion, thereby establishing a positive feedback loop (59). In parallel,CXCL12 upregulates HOXB5 via the ERK/ETS1 pathway, and HOXB5 in turn transcriptionally activates ITGB3 (encoding integrin β3), further amplifying migratory signals (42).Epithelial–mesenchymal transition (EMT) is a biological process in which epithelial cells acquire mesenchymal traits in response to various physiological and pathological stimuli. This process is characterized by decreased E-cadherin expression, increased β-catenin translocation from the membrane to the cytoplasm, and cytoskeletal switching from keratin to vimentin-based filaments (60). In CRC, the CXCL12/CXCR4 axis drives this transformation, thereby reducing basement membrane adhesion and promoting metastasis (61).

CXCR4 regulates the expression of E-cadherin and vimentin,consequently promoting EMT and enhancing the metastatic capacity of CRC cells (61, 62). Concurrently, β-catenin translocates from the membrane to the cytoplasm, reducing the formation of β-catenin/E-cadherin complexes and facilitating EMT (63). Notably, Knockdown of CXCR4 or CXCL12 in HT-29 cells downregulates β-catenin (54), while β-catenin pathway activation enhances CXCR4 expression in HCT116 cells (64), thus establishing a bidirectional regulatory loop that sustains EMT.

Beyond tumor cell-autonomous mechanisms, the CXCL12/CXCR4 axis integrates stromal signals to amplify EMT. CAFs promote EMT through exosomes (65) and activin A (66). Furthermore, CXCL12 derived from hepatic stellate cells (HSCs) and mesenchymal stem cells (MSCs) activates CXCR4 signaling in CRC cells, leading to upregulation of transforming growth factor-β1 (TGF-β1), while also promoting the differentiation of HSCs and MSCs into CAFs (67, 68). Recent studies have further revealed that SPOCD1 enhances CXCL12 expression in CAFs by upregulating LAMA4, which in turn enables CXCL12 to engage CXCR4 on CRC cells and activate EMT signaling, ultimately driving metastasis. Thus, the CXCL12/CXCR4 axis orchestrates EMT through both intrinsic signaling and stromal crosstalk.

Collectively, these findings demonstrate that the CXCL12/CXCR4 axis drives CRC metastasis by integrating chemotactic guidance, ECM-remodeling invasive programs, and EMT-mediated phenotypic plasticity. Through this coordinated regulation, CRC cells can sense CXCL12 gradients, acquire motility, dismantle local tissue barriers, and preferentially disseminate to CXCL12-enriched organs. Therefore, migration and invasion represent one of the most fundamental mechanisms by which the CXCL12/CXCR4 axis mediates site-specific metastasis in CRC.

### 2.2 Promoting vascularization and lymphangiogenesis

Blood vessels supply tumors with essential nutrients and, along with lymphatic vessels, facilitate distant metastasis (69). Consequently, angiogenesis and lymphangiogenesis at the tumor site are indispensable for tumor progression and metastasis. CXCL12/CXCR4 not only drives metastasis of CXCR4-containing tumor cells to lymph nodes through chemotaxis, but also promotes angiogenesis and lymphangiogenesis in tumor microenvironment (TME) by regulating vascular endothelial growth factor (VEGF) and directly chemoattracting endothelial progenitors, thus facilitating CRC metastasis (47, 70).

VEGF-C, produced by various cell types, binds to the VEGFR-3 receptor on lymphatic endothelial cells, thereby stimulating lymphatic vessel formation (71). The CXCL12/CXCR4 axis plays a crucial role in cancer progression by facilitating the movement of CXCR4-expressing tumor cells to lymph nodes (72) and enhancing the motility of lymphatic endothelial cells that also express CXCR4 (73), collectively contributing to lymphatic vessel formation. Of note, VEGF-C further stimulates the production of CXCR4, creating a positive feedback loop that reinforces this pathway (72). This intricate interplay not only supports tumor cell dissemination but also promotes lymphatic remodeling, generating a microenvironment conducive to metastatic spread. Consistent with these findings,Fukunaga et al. demonstrated a direct correlation between VEGF-C levels and CXCR4 expression in the lymph node metastasis of CRC (74).

VEGF promotes angiogenesis and upregulates components of the CXCL12/CXCR4 axis. Conversely,CXCL12 on endothelial cells can also upregulate VEGF expression through paracrine signaling, and these two factors act in concert to facilitate CRC metastasis (75). Hypoxia-inducible factor-1α (HIF-1α) increases CXCR4 expression in CRC under both hypoxic and normoxic conditions (76). Yang et al. reported that high concentrations of Chanling Gao (CLG) can suppress the protein expression of CXCL12, CXCR4, and HIF-1α in metastatic tumors, as demonstrated in mouse xenograft models of CRC. Mechanistically, the high-dose CLG group reduced the expression levels of PI3K, Akt, and VEGF compared to the control group, suggesting that CLG may suppress VEGF production and release through the HIF-1α/CXCL12/CXCR4/PI3K-Akt pathway, thereby suppressing angiogenesis and impeding CRC metastasis (77).

Furthermore, by binding to CXCR4 on vascular endothelial cells, CXCL12 can induce their directional migration toward the tumor site, thereby promoting the development of tumor-associated blood vessels. This process enhances vascularization, supplying tumors with essential nutrients and oxygen for growth while simultaneously facilitating further metastatic spread (47).

### 2.3 Shaping the tumor immune microenvironment

The differential expression of CXCR4 across immune cell subsets is a critical determinant of the immunosuppressive microenvironment in CRC. Tregs express higher levels of CXCR4 on their surface compared to CD8^+^ or CD4^+^ effector T cells, rendering them more responsive to CXCL12 gradients and enabling their preferential recruitment to CXCL12-rich tumor regions (78, 79). Similarly, M2-polarized macrophages and TAMs exhibit elevated CXCR4 expression relative to classically activated M1 macrophages, which contributes to their accumulation in the tumor stroma and sustains their immunosuppressive functions (80, 81). This differential expression pattern explains why CXCL12/CXCR4 signaling preferentially recruits immunosuppressive populations over effector lymphocytes, thereby actively shaping a tolerogenic microenvironment that supports tumor progression and immune evasion.

#### 2.3.1 Recruitment of immunosuppressive populations

CXCL12 serves as a potent chemoattractant for multiple CXCR4-expressing immunosuppressive cell types, actively shaping the cellular composition of the tumor microenvironment. Tregs are recruited along CXCL12 gradients, where they suppress effector T cell functions through contact-dependent mechanisms, cytokine-mediated secretory suppression (e.g., IL-10 and TGF-β), and metabolic reprogramming (e.g., IL-2 consumption) (48, 49, 82). CRC cells upregulate CXCL12 expression in peritumoral fibroblasts through exosomal HIF2A. The CXCL12-CXCR4 axis further promotes the recruitment of monocytes via the MAPK/ERK signaling pathway, which subsequently polarize into M2-like tumor-associated macrophages (TAMs) characterized by elevated expression of CD163 and CD206 and secretion of IL-10 and TGF-β, thereby further enhancing the immunosuppressive microenvironment (80). In addition, activation of the CXCL12/CXCR4 axis upregulates miR-25-3p, miR-130b-3p, and miR-425-5p in CRC cells, which can be transferred to macrophages via exosomes. These exosomal microRNA (miRNAs) induce M2 polarization of macrophages through the PTEN/PI3K/Akt pathway, thereby enhancing EMT and the secretion of vascular endothelial growth factor (VEGF), ultimately promoting cancer metastasis (81).

Myeloid-derived suppressor cells (MDSCs) effectively inhibit the activity of cytotoxic T lymphocytes and natural killer cells. These cells also express CXCR4 and accumulate within CXCL12-rich tumor regions (61, 83, 84). Yu et al. demonstrated using villin-CXCR4 transgenic mice that CXCR4 overexpression significantly promotes the infiltration of granulocytic MDSCs (G-MDSCs, CD11b+Ly6G+) and monocytic MDSCs (M-MDSCs, CD11b+Ly6C+) into colonic tissue, thereby accelerating colitis-associated and Apc mutation-driven colorectal tumorigenesis (61). Importantly, CXCR4 blockade with AMD3100 abrogated this effect, highlighting the dependency of MDSC recruitment on CXCR4 signalin (61). Mechanistically, CXCR4 activation induced the upregulation of lncRNA XIST, which functions as a ceRNA to sponge miR-133a-3p, ultimately promoting cytoskeletal remodeling and tumor cell migration (61). In this study, investigators used flow cytometry to analyze peripheral blood, peritumoral, and tumor tissues from 34 patients with colorectal CRC liver metastasis (CRLMs). The results demonstrated that M-MDSCs (CD14^+^HLA-DR^neg^/^low^CD15^-^) were significantly elevated, while PMN-MDSCs (CD11b^+^CD15^+^CD33^+^Lin^-^HLA-DR^neg^/^low^) showed no obvious increase, and the degree of MDSC-related immunosuppression was weaker in CRLMs than in hepatocellular carcinoma (85). however, Zhao et al. reported an apparently paradoxical observation in CRC: CXCL12 was downregulated in tumor tissues, and silencing CXCL12 unexpectedly promoted CRC cell migration and invasion. Flow cytometry revealed that CXCL12 was negatively correlated with the proportion and absolute number of both M-MDSCs (CD14^+^HLA-DR^low^/CD11b^+^) and G-MDSCs (CD15^+^CD11b^+^HLA-DR^low^); CXCL12 silencing markedly promoted the accumulation of these two MDSC subsets, whereas CXCL12 overexpression combined with anti-PD-L1 antibody significantly reduced the levels of both M-MDSCs and G-MDSCs and suppressed CRC cell proliferation, migration and invasion (86). This finding stands in stark contrast to the traditional view that the CXCL12/CXCR4 axis recruits MDSCs, and the underlying mechanism warrants further investigation.

Furthermore, exosomes play a crucial role in CXCL12/CXCR4-mediated MDSC recruitment during pre-metastatic niche formation. Studies have shown that high PRL-3 expression in CRC cells upregulates integrin αvβ5 in secreted exosomes via the Src/STAT3 signaling pathway. These exosomes specifically target F4/80^+^ macrophages in the liver, activating the p38/STAT1 signaling pathway, which in turn enhances macrophage secretion of CXCL12 to recruit MDSCs and establish the hepatic pre-metastatic niche (83). In addition, CRC cells remotely regulate the hepatic pre-metastatic microenvironment through the lncRNA MIR181A1HG carried in exosomes. After being packaged into exosomes by HNRNPA2B1, this lncRNA is taken up by HSCs, where it functions as a ceRNA by sponging miR-373-3p. This derepresses TGFBRII, activating the TGF-β/Smad2/3 pathway and subsequently activating HSCs. These activated HSCs then secrete CXCL12, which promotes liver metastasis by recruiting MDSCs (84). The recruitment of these diverse immunosuppressive cell populations fosters a tolerogenic microenvironment that shields developing metastases from immune surveillance.

#### 2.3.2 Exclusion and dysfunction of effector lymphocytes

In colorectal cancer, the CXCL12/CXCR4 axis regulates T cells primarily by inhibiting their infiltration into tumors and modulating their effector functions. This axis globally suppresses T cell infiltration into the tumor through a “chemokine receptor interference” mechanism. Biasci et al., using human immune cell chemotaxis assays, found that CXCL12-stimulated CXCR4 significantly inhibits chemotaxis mediated by CXCR3, CXCR5, CXCR6, and CCR2, receptors that are differentially expressed on key effector cell subsets such as CD8^+^ T cells, CD4^+^ T cells, and tissue-resident memory T cells (49).

Furthermore, senescent tumor cells construct a “cytokine barrier” via the CXCL12/CXCR4 axis to directly inhibit CD8^+^ T cell function.While CXCL12 can attract T cells at low physiological concentrations, the high local concentrations frequently observed in the tumor microenvironment induce CXCR4 internalization and degradation on CD8^+^ cytotoxic T lymphocytes, rendering them unable to respond to chemotactic cues (87).

In addition, the CXCL12/CXCR4 signaling axis can form a physical barrier against T cells. Wang et al., through analysis of CRC patient samples resistant to PD-1 immunotherapy combined with *in vitro* cell experiments, discovered that extracellular CXCL12 can covalently bind to keratin-19 (KRT19) on the surface of tumor cells, forming a filamentous network structure. This covalent binding process is catalyzed by transglutaminase 2 (TGM2), which forms isopeptide bonds between KRT19 and CXCL12, stably connecting the two and assembling them into a filamentous complex coating that envelops tumor cells. This CXCL12-KRT19 complex, through high local concentrations of CXCL12, induces CXCR4 internalization and chemotactic dysfunction on T cell surfaces, creating both physical and functional immune barriers that effectively exclude CD8^+^ T cell tumor infiltration (88).

In CMS4 CRC, CAFs secrete CXCL12, which recruits bone marrow-derived, CXCR4-expressing monocyte-like cells into the tumor. These cells highly express THBS1 (thrombospondin-1), a matricellular protein with dual functions: it directly inhibits CD8+ T cell activation and induces their conversion to an exhausted state via the CD47 and CD36 pathways (89), while also serving as a potent anti-angiogenic factor that suppresses tumor vascularization (90). In addition, CAF-derived CXCL12 promotes the retention of neutrophils within the tumor by inducing a CXCR2^+^CXCR4^+^ double-positive phenotype. These retained CD15^high^ neutrophils interact directly with CD8^+^ T cells, inducing their differentiation into Granzyme K^high^ (GZMK^high)^ CD39^−^ effector memory T cells (TEM). The co-localization of these cells upregulates GZMK, which is then released and acts in a non-cytotoxic manner on intestinal epithelial cells, downregulating E-cadherin and promoting EMT, thereby driving tumor progression and recurrence (91).

In colorectal cancer, the CXCL12/CXCR4 axis regulates natural killer (NK) cells primarily by modulating their tumor infiltration and functional status. Studies have demonstrated that during fasting-induced Natural Killer Cell (NK cell) redistribution, the relocation of NK cells to the bone marrow is dependent on CXCR4, highlighting the pivotal role of this axis in regulating the tissue localization of NK cells (92). Consistent with this, clinical research has shown that CD56^bright^ NK cells in CRLMs highly express CXCR4, and blocking CXCR4 enhances their ability to produce Interferon-gamma (IFN-γ) and Tumor Necrosis Factor Alpha (TNF-α), suggesting that this axis negatively regulates NK cell function (93).Mechanistic studies further indicate that the CXCL12/CXCR4 axis not only mediates chemotaxis but also induces p42 phosphorylation and calcium influx, leading to the depletion of NK cell degranulation reserves and desensitization to subsequent tumor stimulation. Moreover, this axis synergizes with S1P5 to inhibit NK cell function (94). In a mouse model of prostate cancer, CXCL12-CXCR4 interaction was shown to activate the transcription factor C/EBPβ within NK cells, leading to dysregulation of the autophagy pathway. This directly results in mitochondrial dysfunction and loss of effector function; however, whether this mechanism operates in CRC remains to be elucidated (95). Collectively, the CXCL12/CXCR4 axis bidirectionally regulates NK cell function in CRC.Therefore, targeting this axis holds promise for enhancing anti-tumor immunity by abrogating its inhibitory signals.

In colorectal cancer, the CXCL12/CXCR4 axis regulates neutrophils primarily by governing their bone marrow egress, tumor infiltration, and contribution to therapy resistance. First, this axis plays a pivotal role in regulating the release of neutrophils from the bone marrow, with CXCR4 signaling maintaining their retention within this niche (91, 96). Second, CXCL12 gradients within the tumor microenvironment can attract CXCR4^+^ neutrophils to infiltrate the tumor site. Specifically,utilizing a CRC mouse model, Jung et al. demonstrated that anti-VEGFR2 therapy upregulates CXCL12/CXCR4 axis expression in tumor tissue, which subsequently recruits immunosuppressive Ly6G^+^ neutrophils, mediating resistance to anti-angiogenic therapy (97). Conversely, blocking CXCR4 with the inhibitor AMD3100 significantly reduces the infiltration of these cells and restores the efficacy of anti-angiogenic therapy (97). In summary, the CXCL12/CXCR4 axis plays a critical role in CRC progression and therapy resistance by regulating the bone marrow egress and tumor infiltration of neutrophils.

Currently, there are few studies on the mechanism of the CXCL12/CXCR4 axis in relation to dendritic cells, B cells, mast cells and other cells in colorectal cancer. The CXCL12/CXCR4 axis plays an important role in the immune microenvironment of CRC by regulating the tumor infiltration of these cells. Through human immune cell chemotaxis assays, Biasci et al. found that CXCR4 stimulated by CXCL12 can significantly inhibit the chemotaxis mediated by CXCR1, CXCR3, CXCR5, CXCR6 and CCR2, and these receptors are expressed on the surface of various immune cells including B cells and dendritic cells (49). The functional impact of the CXCL12/CXCR4 axis remains to be further investigated.

#### 2.3.3 chronic inflammation

**Figure 1:**
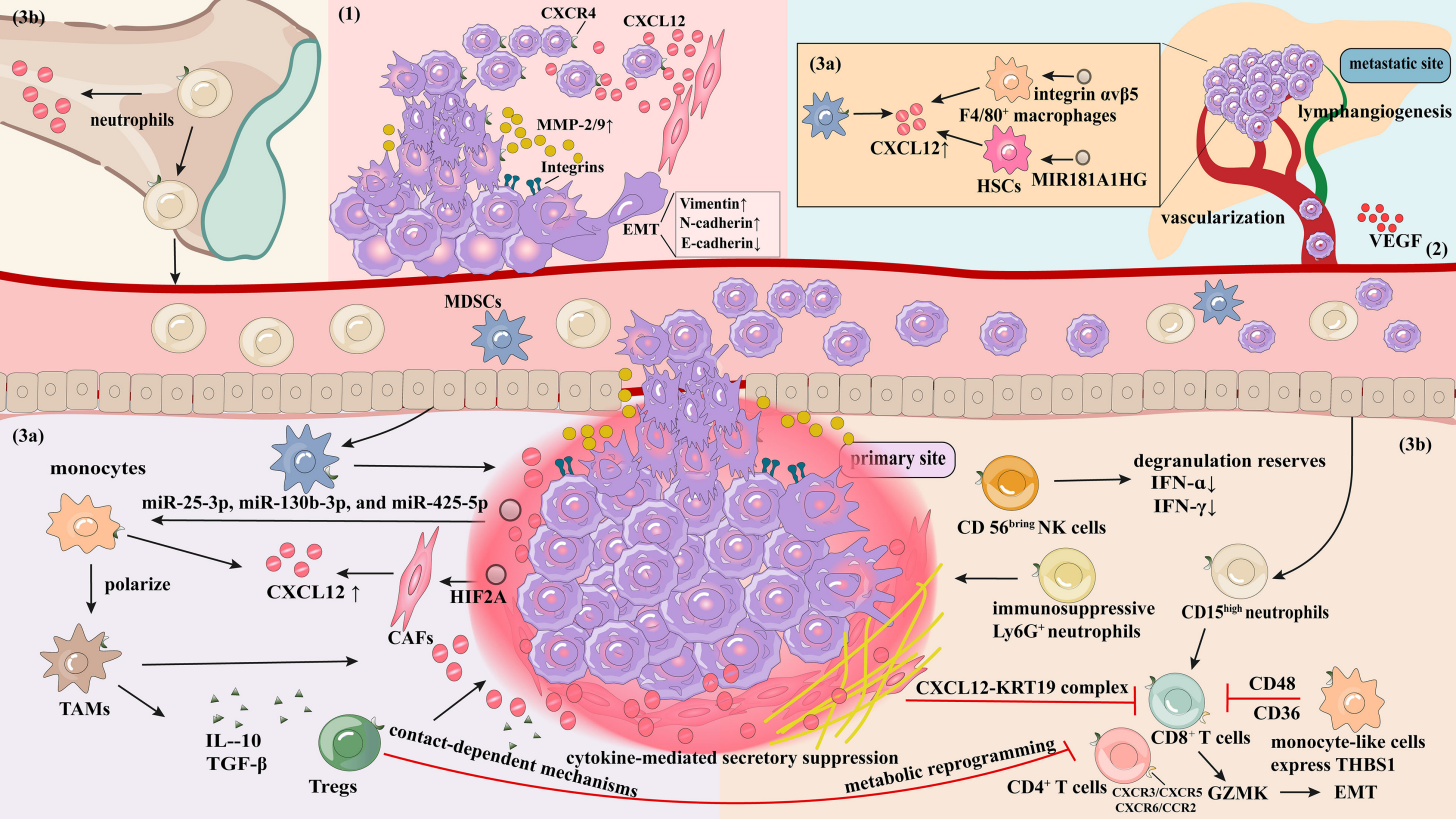
Schematic overview of the CXCL12/CXCR4 axis in CRC metastasis. This schematic illustrates the complex network through which the CXCL12/CXCR4 axis promotes CRC progression and metastasis.

Chronic inflammation plays a central role in CRC initiation and progression by inducing genetic mutations, activating pro-oncogenic pathways, remodeling the microenvironment, and impairing immune surveillance. The CXCR4/CXCL12 axis serves as a bridge connecting inflammation and cancer, exerting multiple regulatory functions. Chronic inflammation can upregulate CXCR4 expression through hypoxia and inflammatory mediators, although the underlying mechanisms in CRC remain relatively understudied (98, 99). Using a villin-CXCR4 transgenic mouse model in an azoxymethane/dextran sulfate sodium (AOM/DSS)-induced colitis-associated cancer model, Yu et al. demonstrated that CXCR4 overexpression significantly increased tumor burden. Furthermore, CXCR4^+^/^-^Apc^min^/^+^ compound mutant mice exhibited higher tumor incidence compared to Apc^min^/^+^ mice alone, confirming that CXCR4 accelerates Apc mutation-driven tumorigenesis. Mechanistically, CXCR4 promotes EMT and the infiltration of immunosuppressive cells (macrophages and MDSCs),while upregulating lncRNA XIST, which acts as a ceRNA to sponge miR-133a-3p, thereby derepressing its target gene RhoA and activating the RhoA/ROCK/p-MLC pathway to promote cytoskeletal reorganization and tumor cell migration (61). In a colitis-associated carcinogenesis model, Wu et al. found that AMD3100 promotes M1 polarization of peritoneal macrophages, reduces their recruitment activity, and decreases serum levels of IL-12 and IL-23 (100). In summary, chronic inflammation drives overactivation of the CXCR4/CXCL12 axis, while the activation of this axis in turn amplifies immunosuppression, forming a positive feedback loop that tightly links chronic inflammation to tumor progression (**Figure 1**).

1. Driving migration and invasion: CXCR4-high CRC cells migrate along the CXCL12 concentration gradient; CXCL12 binding to CXCR4 on CRC cells activates ECM degradation via MMP-2/9 and integrins; CXCL12/CXCR4 signaling axis induces EMT characterized by E-cadherin loss and vimentin gain.
2. Angiogenesis and lymphangiogenesis CXCR4 activation promotes vascularization and lymphangiogenesis through VEGF/VEGFR signaling and endothelial progenitor cell recruitment, providing conduits for systemic dissemination and nutrient supply.
3. Tumor immune microenvironment remodeling.
  - Recruitment of immunosuppressive populations:In the CRC immune microenvironment, CAFs secrete CXCL12, recruiting major CXCR4-expressing immunosuppressive cells (Tregs, MDSCs, TAMs, immunosuppressive Ly6G^+^ neutrophils) to the tumor periphery. CRC cells enhance CAF-derived CXCL12 production via exosomal HIF2A, promoting recruitment of CXCR4-expressing monocytes that subsequently differentiate into TAMs. Additionally, CRC cells transfer exosomal miR-25-3p, miR-130b-3p, and miR-425-5p to monocytes, inducing TAM polarization. TAMs secrete IL-10 and TGF-β. Tregs suppress effector T cell functions (e.g., CD4^+^ and CD8^+^ T cells) through contact-dependent mechanisms, cytokine-mediated suppression (IL-10 and TGF-β), and metabolic reprogramming. During pre-metastatic niche formation, tumor cells utilize exosomal integrin αvβ5 and the non-coding RNA MIR181A1HG to stimulate F4/80^+^ macrophages and HSCs, respectively, promoting CXCL12 secretion and subsequent recruitment of CXCR4-expressing MDSCs.
  - Exclusion and dysfunction of effector lymphocytes: The CXCL12/CXCR4 axis globally suppresses T cell infiltration through “chemokine receptor interference,” inhibiting chemotaxis mediated by CXCR3, CXCR5, CXCR6, and CCR2. A “cytokine barrier” formed by high local CXCL12 concentrations induces CXCR4 internalization and degradation on CD8^+^ T cells, directly impairing their chemotaxis. Physical exclusion of CD8^+^ T cells is achieved through CXCL12-KRT19 filamentous complexes. The axis recruits monocyte-like cells highly expressing THBS1 (thrombospondin-1), which inhibit CD8^+^ T cell activation and induce exhaustion via CD47 and CD36 pathways. CXCL12 retains CXCR4-expressing neutrophils in the bone marrow and tumors; tumor-retained CD15high neutrophils induce CD8^+^ T cell differentiation into GZMK^high^ CD39− effector memory T cells (TEM), with released GZMK downregulating E-cadherin and promoting EMT in intestinal epithelial cells. The CXCL12/CXCR4 axis recruits NK cells, depleting their degranulation reserves and negatively regulating IFN-γ and TNF-α production by CD56^bright^ NK cells. During pre-metastatic niche formation, tumor cells utilize exosomal integrin αvβ5 and MIR181A1HG to stimulate F4/80^+^ macrophages and HSCs, respectively, promoting CXCL12 secretion and MDSC recruitment.

## 3 Molecular mechanisms of CXCL12/CXCR4 metastasis in CRC

### 3.1 Ligand-receptor interaction and signaling initiation

The N-terminal region of CXCL12 interacts with amino acid residues on the extracellular side of the second and third transmembrane helices of CXCR4, whereas the core domain of CXCL12 binds to the N-terminus of CXCR4. This dual-site binding induces a conformational change in the receptor structure, thereby enhancing the binding affinity and specificity of the chemokine-receptor interaction (101). Concurrently, intracellular guanine nucleotide exchange occurs, wherein GDP is replaced by GTP, leading to the dissociation of heterotrimeric G proteins into α subunits bound to βγ dimers and GTP. This activation initiates downstream signaling, including the PI3K/Akt, Wnt/β-catenin, and Ras/Raf/MEK/ERK pathways (102, 103).

In addition to this classical interaction, CXCL12 binds with high affinity to CXCR7, an atypical chemokine receptor (104, 105). CXCR7 contains an altered DRY motif in its intracellular loops, rendering it structurally incapable of activating classical G protein signaling (106). Instead, ligand binding induces specific phosphorylation of the receptor by G protein-coupled receptor kinases (GRKs), leading to biased β-arrestin-mediated signaling (106). CXCR7 primarily functions as a scavenger receptor, internalizing and degrading extracellular CXCL12, thereby modulating its availability of CXCL12 in the tumor microenvironment (105, 107, 108). This activity establishes and maintains a CXCL12 concentration gradient that guides the migration and invasion of CXCR4-expressing tumor cells, while simultaneously preventing CXCR4 desensitization from sustained stimulation and finely tuning CXCR4-mediated signaling (107, 108). In colorectal cancer, CXCR7 expression is significantly upregulated and correlates with metastasis and poor prognosis (105).

Notably, CXCR7 can form heterodimers with CXCR4, altering downstream signaling preferences, thereby increasing the functional complexity of the CXCL12 axis. Decaillot et al. first demonstrated that upon co-expression with CXCR4, the heterodimeric complex constitutively recruits β-arrestin while simultaneously attenuating G protein-mediated signaling, resulting in a shift from the classical G protein pathway to β-arrestin-dependent MAPK pathways (including ERK1/2, p38 MAPK) (109). Extending these findings to colorectal cancer, Song et al. showed that the CXCR7/CXCR4 heterodimer promotes tumorigenesis by inducing β-arrestin 1 nuclear translocation and recruiting the histone demethylase JMJD2A, thereby upregulating the transcription of pro-inflammatory factors and oncogenes (110).Beyond heterodimerization with CXCR7, CXCR4 can also form complexes with CCR2, which promotes the recruitment of both G proteins and β-arrestin, resulting in a unique synergistic effect on calcium signaling (111). In contrast, CXCR4 stimulated by CXCL12 actively cross-inhibits the signal transduction mediated by CCR2, CXCR1, CXCR3, CXCR5, and CXCR6, thereby disrupting the directional migration of the monocyte lineage (49).While receptor heterodimerization adds another layer of regulatory complexity to CXCL12/CXCR4 signaling, the specific mechanisms underlying these interactions in CRC require further validation.

In addition to CXCL12, other chemokines and inflammatory mediators have been identified as ligands for CXCR4. MIF can directly bind to CXCR4 and activate downstream signaling pathways, including the MAPK cascade (112). In CRML, single-cell transcriptomic analysis has revealed that the interaction between MIF and its receptors CD74 and CXCR4 is significantly enhanced during metastasis progression, thereby promoting tumor cell invasion and migration (113, 114). Furthermore, HMGB1 has been shown to interact with CXCR4 and trigger downstream signaling pathways involved in inflammation and tumor progression, although this mechanism requires further validation in CRC (115).

### 3.2 Canonical downstream signaling pathways

#### 3.2.1 PI3K/Akt

The PI3K/Akt signaling pathway is one of the predominant downstream routes of the CXCL12/CXCR4 axis, a conclusion supported by pan-cancer studies (116). In pancreatic cancer and renal cell carcinoma, the rapid proliferation and metastasis of cancer cells are closely linked to this signaling pathway (117, 118). Collectively, these findings establish the PI3K/Akt pathway as a central mediator of CXCL12/CXCR4-driven CRC progression. Upon binding to its receptor CXCR4, CXCL12 activates PI3K, which subsequently phosphorylates PIP2 to PIP3 at the cell membrane (56). Akt and PDK1 are recruited to the plasma membrane by PIP3, where PDK1 phosphorylates Akt (116). Phosphorylated Akt then promotes CRC cell growth and spread by inducing specific downstream effectors (56).

Activated Akt phosphorylates numerous downstream effectors that collectively drive CRC progression. These include mTOR (enhancing protein synthesis and cell growth), FOXO (inhibiting apoptosis), NF-κB (mediating anti-apoptotic, pro-inflammatory and pro-tumorigenic gene transcription) and GSK3β (inactivating this kinase to stabilize β-catenin) (119). Phosphatase and tensin homolog deleted on chromosome ten (PTEN) is a tumor suppressor that encodes a phosphatase which directly dephosphorylates PIP3 to PIP2, thereby attenuating PI3K/Akt signaling (120).

#### 3.2.2 Wnt/β-catenin

The Wnt/β-catenin pathway is a highly conserved signaling cascade that regulates diverse cellular processes. In the absence of pathway activation, β-catenin is phosphorylated by GSK3 and CK1 and ubiquitinated by β-TrCP within the destruction complex, comprising Dvl, Axin, CKI, APC, GSK3, β-catenin, and β-TrCP, targeting it for proteasomal degradation (121). Pathway activation is initiated upon Wnt binding to the Frizzled receptor, which recruits Daple and promotes Dvl phosphorylation, thereby preventing β-catenin phosphorylation and ubiquitination within the destruction complex (122). β-catenin accumulation in the cytoplasm and its subsequent nuclear translocation activate the transcription factors TCF and LEF, leading to the transcription of downstream target genes that drive cell proliferation, survival, and migration (123).

The CXCL12/CXCR4 axis intersects with Wnt/β-catenin signaling at multiple levels. Yoon et al. recently demonstrated that the triazolyl-quinoxaline small molecule SRN-18 exerts anti-EMT effects in CRC cells by suppressing the Wnt/β-catenin pathway, which leads to downregulation of CXCR4 and CXCR7 expression along with their downstream signaling proteins including NF-κB and JNK (124). This finding confirms that Wnt/β-catenin signaling acts upstream of CXCR4, regulating its expression at the transcriptional level. Conversely, Mei et al. demonstrated that miR-1269a-mediated repression of Protocadherin gamma subfamily A, 9 (PCDHGA9) releases Homeobox A1 (HOXA1), which translocates to the nucleus, binds to the CXCR4 promoter, and activates its transcription, thereby upregulating CXCR4 and activating the Wnt/β-catenin pathway (125). The reciprocal regulation between CXCR4 and Wnt/β-catenin establishes a bidirectional regulatory loop that sustains both pathways and amplifies pro-metastatic signaling (54, 64).Wang et al. demonstrated that CXCL12/CXCR4 activation in HT-29 colon cancer cells reduces E-cadherin expression and destabilizes the β-catenin complex, correlating with enhanced cell migration (126). While Zhang et al. reported in cholangiocarcinoma that PI3K/Akt signaling suppresses kinase 3β(GSK3β) activity, leading to β-catenin stabilization and Wnt pathway activation (127), this mechanism remains to be directly validated in CRC. Specifically, whether CXCL12/CXCR4 induces GSK3β degradation (e.g., via ubiquitination) in CRC, rather than mere inactivation, requires further investigation through proteomic or CRISPR-based studies.

#### 3.2.3 Ras/Raf/MEK/ERK

The Ras–Raf–MEK1/2–ERK pathway, a major branch of MAPK signaling, can be activated downstream of multiple cell-surface receptors, including receptor tyrosine kinases and G protein-coupled receptors such as CXCR4. Pathway activation is initiated upon binding of extracellular stimuli to receptor tyrosine kinases (RTKs) at the cell membrane, leading to the recruitment and activation of Ras. Activated Ras subsequently engages Raf, which then phosphorylates and activates MEK1/2, culminating in ERK phosphorylation. Phosphorylated ERK translocates to the nucleus, where it regulates transcription factors—including Ets-1, Elk-1, and c-Myc—that control the expression of genes involved in cell proliferation, survival, and apoptosis (127–131).

Multiple lines of evidence link the Ras-ERK pathway to CXCR4 signaling in CRC. Urosevic J et al. demonstrated in SW620 cell cultures and mouse models that CXCR4 activated the Ras-ERK1/2 pathway and increased IL-10 and CXCL1 expression. Conversely, Ras-ERK1/2 upregulates CXCR4 expression, thereby establishing a positive feedback loop that promotes CRC progression (50).

Adding another layer of complexity, Feng et al. demonstrated that CXCL12 activates the ERK pathway, which upregulates HOXB5. HOXB5 then binds to the CXCR4 promoter and transactivates its expression, forming a reciprocal positive feedback loop that amplifies migratory signals (42).

### 3.3 Multilayered regulation of CXCL12/CXCR4 expression and function

Both CXCR4 and its ligand CXCL12 are regulated at multiple levels, including transcriptional, post-transcriptional, translational, epigenetic, and post-translational mechanisms. These regulatory layers dynamically shape the activity of the CXCL12/CXCR4 axis within the CRC tumor microenvironmen. While the regulation of CXCR4 has been more extensively characterized, emerging evidence indicates that CXCL12 is also subject to complex control by hypoxia, stromal signaling, non-coding RNAs, DNA methylation, and proteolytic processing.

#### 3.3.1 Transcriptional regulation

The constitutive and inducible expression of CXCR4 is profoundly shaped by the TME through the action of specific transcription factors. Of note, the constitutive expression of CXCR4—observed across most tumor cell types, including colorectal cancer—is a fundamental characteristic that enables cancer cells to respond to CXCL12 gradients present in distant metastatic sites (132). This property, rooted in CXCL12’s nature as a homeostatic chemokine, underlies the ability of CXCR4-expressing tumor cells to engage in organ-specific metastasis, as originally proposed by Zlotnik (12, 133).

HIF-1α is one of the most critical activators. In the hypoxic core of solid tumors such as CRC, stabilized HIF-1α translocates to the nucleus and binds to hypoxia-response elements within the CXCR4 promoter. This targeted transcriptional activation drives robust upregulation of CXCR4, thereby promoting tumor cell migration toward oxygen- and nutrient-rich metastatic microenvironments (76, 98).

Additionally,nuclear factor-κB (NF-κB) contributes to CXCR4 transcriptional activation. In inflammatory microenvironments, activation of the NF-κB signaling pathway directly upregulates CXCR4 expression, linking inflammatory signals to the chemokine axis.

Furthermore, CXCR4 transcription is governed by a self-amplifying positive feedback loop. Recent experimental evidence indicates that CXCL12 stimulation activates the homeobox transcription factor HOXB5, which then binds directly to the CXCR4 promoter and further transactivates its expression. This autonomous CXCL12–HOXB5–CXCR4 positive feedback loop locks CRC cells in a highly invasive state (42). HOXA1 transactivates CXCR4 expression, thereby activating the downstream Wnt/β-catenin pathway and promoting CRC metastasis (125).

The expression of CXCL12 is directly regulated by transcription factors that respond to microenvironmental cues. Under hypoxic conditions, HIF-1α binds to hypoxia-response elements within the CXCL12 promoter, driving its transcriptional upregulation and establishing chemotactic gradients that guide progenitor cell migration (134). Direct evidence from dual-luciferase reporter assays has confirmed that HIF-1α binds to the HBS1 site in the CXCL12 promoter within cancer-associated fibroblasts (CAFs) associated with colorectal cancer liver metastases (CRLM), thereby transcriptionally activating CXCL12 expression (135).

Beyond hypoxia, tumor-derived exosomal miR-146a-5p and miR-155-5p activate CAFs via the JAK2-STAT3/NF-κB signaling pathway, promoting CXCL12 secretion, which in turn induces epithelial–mesenchymal transition (EMT) and enhances the metastatic potential of CRC cells [24]. Furthermore, the SPOCD1–LAMA4 axis has been identified as a critical upstream regulator of CXCL12 expression in CAFs: tumor cell-derived SPOCD1 upregulates LAMA4 via DNMT1-mediated DNA methylation, which subsequently enhances CXCL12 transcription in CAFs, establishing a feedforward loop that amplifies CXCR4-dependent signaling in CRC cells (44).

Of note, despite the established role of HIF-1α in CXCL12 regulation in other cancer types, direct evidence supporting hypoxia-driven CXCL12 upregulation specifically in CRC remains limited, warranting further investigation.

#### 3.3.2 Post-transcriptional and translational regulation

In addition to transcriptional control, CXCR4 expression is also regulated at the post-transcriptional and translational levels. These regulatory processes are mediated by non-coding RNAs, RNA-binding proteins, and context-dependent stress responses within the tumor microenvironment. Among them, miRNAs represent one of the best-characterized mechanisms, as they suppress gene expression by promoting mRNA degradation and/or inhibiting translation of target transcripts (136). Through these effects, miRNAs can fine-tune CXCR4 protein expression and thereby influence CRC cell migration, invasion, metastatic potential, and treatment response.

MicroRNAs, a class of non-coding RNAs, play a crucial role in CRC development and progression. Several miRNAs closely associated with CRC cell behavior have been identified. For instance, miR-126 (137) and miRNA-766 (138) are downregulated in CRC cells, yet can target and suppress the highly expressed CXCR4, thereby inhibiting CRC cell migration.

Beyond miRNAs that directly suppress CXCR4, some miRNAs are themselves regulated by the CXCL12/CXCR4 axis. Shirafkan et al. demonstrated that miR-193a-5p downregulates CXCR4 expression in CRC cells, reducing their migratory capacity *in vitro* using HT-29 cells (139).Yu et al. investigated the inhibitory mechanism of the CXCL12/CXCR4 axis on miR-133a-3p expression in HCT116 and SW620 cells.They found that the CXCL12/CXCR4 axis induced the expression of the lncRNA XIST, which bound to miR-133a-3p and competitively inhibited its binding to the target gene RhoA. This process, termed ‘sponging’ of miR-133a-3p, increased RhoA expression and contributed to CRC cell metastasis (61).

Adding further complexity, miR-125b exhibits bidirectional regulation with the CXCL12/CXCR4 axis: it is not only induced by this axis but also promotes CXCR4 expression, forming a positive feedback loop that exacerbates CRC metastasis (140). Additionally, miR-125b enhances autophagy in CRC cells, conferring resistance to 5-fluorouracil (5-FU)-induced apoptosis and contributing to chemoresistance (140).

DNA methylation plays a critical role in silencing CXCL12 expression. In colorectal cancer, hypermethylation of the CXCL12 promoter leads to transcriptional silencing, and this loss of CXCL12 expression paradoxically promotes metastasis by disrupting the chemokine retention signal that normally maintains tumor cells at the primary sit (141). Conversely, treatment with Inhibition of DNA methyltransferase (Dnmt) enzymes with 5-aza-2’-deoxycytidine or genetic ablation of both Dnmt1 and Dnmt3b can restore CXCL12 expression, suggesting potential therapeutic avenues (141). Beyond DNA methylation, non-coding RNAs also regulate CXCL12 abundance. Using multi-omics and single-cell approaches, Cheon et al. demonstrated that both miRNA interference and DNA methylation contribute to the reduced CXCL12 expression observed in stromal cells of colon adenocarcinoma (142). Additionally, 5-FU-resistant CRC cells secrete exosomes containing Transient receptor potential cation channel subfamily C member 5 (TRPC5), which induce CAFs to produce increased levels of CXCL12, thereby contributing to chemoresistance and an immunosuppressive microenvironment (143).

#### 3.3.3 Post-translational modifications

As a G protein-coupled receptor (GPCR), CXCR4 function is precisely regulated by multiple post-translational modifications.Phosphorylation represents one of the major regulatory mechanisms: ligand binding induces GRKs (particularly GRK2/3 and GRK6) to phosphorylate the serine/threonine clusters in the intracellular C-terminal tail of CXCR4, promoting β-arrestin recruitment and thereby mediating receptor internalization and signal desensitization (144). Ubiquitination governs the degradation fate of CXCR4. Following ligand stimulation, E3 ubiquitin ligases (such as AIP4) catalyze polyubiquitination of the intracellular domain, targeting it for lysosomal degradation and preventing signal dysregulation caused by sustained receptor activation (145).Crosstalk between ubiquitination and phosphorylation collectively determines the cell-surface expression level and signaling duration of CXCR4.In addition, CXCR4 can undergo tyrosine sulfation at its N-terminus, which is critical for high-affinity binding to CXCL12 (146).

Protein–protein interactions add another layer of complexity to CXCR4 functional modulation. Recent studies have revealed that nucleolin (NCL), a multifunctional nucleolar protein frequently overexpressed on the surface of tumor cells, can directly interact with CXCR4 and enhance pro-metastatic signaling. Using a non-SELEX aptamer screening strategy, researchers identified a novel DNA aptamer, HY-4, which specifically targets NCL with high affinity. Mechanistic investigations demonstrated that HY-4 disrupts the NCL–CXCR4 interaction, significantly inhibits migration and invasion in LoVo CRC cells, and blocks downstream signaling pathways associated with cytoskeleton remodeling (147).

CXCL12 is synthesized as a precursor protein that undergoes proteolytic processing to generate mature, biologically active isoforms. In CRC treatment, chemotherapeutic agents upregulate CD26/DPPIV activity, thereby accelerating N-terminal degradation of CXCL12. This disrupts the CXCL12 chemotactic gradient and suppresses CXCR4-mediated CRC cell migration (148). Furthermore, CXCL12 abundance is dynamically modulated by the tumor microenvironment in response to therapeutic stress. In CRC patients, CXCL12 is downregulated in primary lesions but can be re-induced in residual tumors after radiotherapy or chemotherapy, suggesting that treatment-induced stress reshapes the chemokine landscape to promote recurrence and metastasis (29). Whereas receptor regulation has been more extensively characterized, the mechanisms controlling CXCL12 expression in CRC tumor cells themselves remain comparatively less well defined.

**Figure 2:**
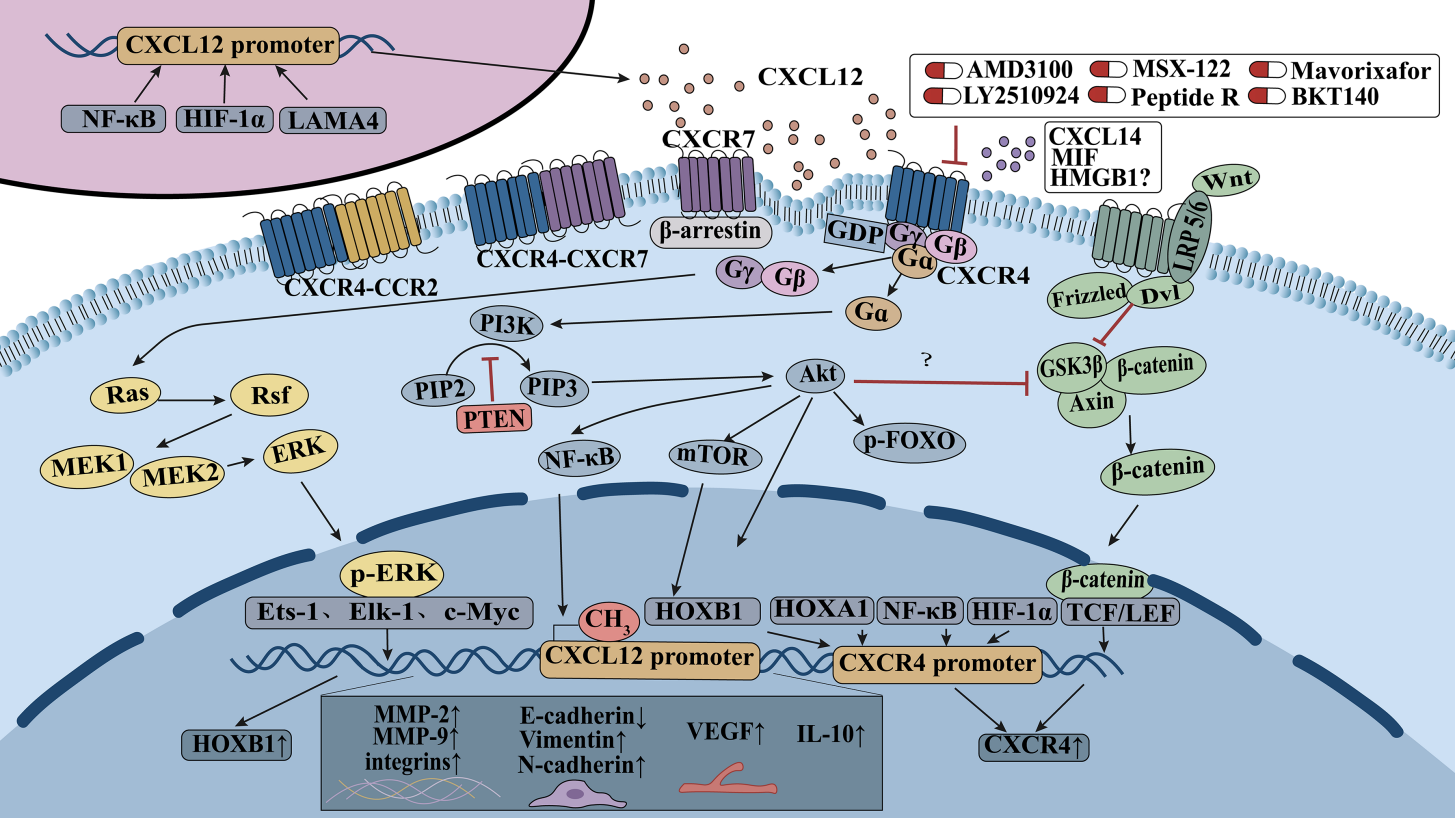
Molecular mechanisms of the CXCL12/CXCR4 signaling axis involved in CRC metastasis.

CXCL12 is primarily derived from CAFs, and its promoter is regulated by NF-κB, LAMA4, and HIF-1α (**Figure 2**). In CRC, CXCL12 is downregulated due to DNA methylation. Upon binding of CXCL12 and other ligands, CXCR4 activates heterotrimeric G-protein signaling and multiple downstream pathways, mainly including the PI3K/Akt, Ras/Raf/MEK/ERK, and Wnt/β-catenin pathways. These signaling cascades promote CRC cell survival, proliferation, migration, invasion, epithelial–mesenchymal transition, angiogenesis, and remodeling of the tumor microenvironment. In addition, downstream transcription factors regulate metastasis-related genes, including MMP-2, MMP-9, integrins, E-cadherin, vimentin, N-cadherin, VEGF, IL-10, and CXCR4. Among them, NF-κB, HIF-1α, HOXA1, and HOXB1 can bind to the CXCR4 promoter and enhance CXCR4 expression. The figure also illustrates the heterodimerization of CXCR4 with CXCR2 and CXCR7, as well as the mechanisms of representative CXCR4-targeting agents.

## 4 Antagonism of CXCL12/CXCR4

Metastasis and drug resistance remain major challenges in CRC treatment. Given the pivotal role of the CXCL12/CXCR4 axis in CRC metastasis, therapeutic targeting of this axis represents a promising strategy. Over the past two decades, multiple CXCR4 antagonists have been developed and evaluated in preclinical and clinical settings, including small molecule inhibitors, peptide antagonists, and monoclonal antibodies.

Plerixafor (AMD3100) is the most extensively studied CXCR4 antagonist. AMD3100 specifically binds to CXCR4 via interaction between its protonated nitrogen. The FDA has approved plerixafor for hematopoietic stem cell mobilization rather than for CRC treatment; nevertheless, it remains the most widely used tool for preclinical CXCR4 inhibition research (149). AMD3100 specifically binds to CXCR4 via interaction between its protonated nitrogen and the receptor’s carboxylate groups (150), thereby antagonizing CXCL12/CXCR4 signaling and inhibiting downstream effectors such as MMP-2 and VEGF (57). It also blocks upstream signals from HIF-1α and HOXB5 to the CXCL12/CXCR4 axis (42, 151).

Peptide R, a novel peptide CXCR4 antagonist, has shown promising preclinical efficacy in CRC. Using HCT116 xenograft mouse models, D’Alterio et al. demonstrated that Peptide R combined with chemotherapy (5-fluorouracil + oxaliplatin) reduced tumor volume by 4-fold after two weeks of treatment, whereas chemotherapy alone or Peptide R alone reduced tumor volume by only 2-fold and 1.6-fold, respectively. Mechanistically, Peptide R reversed chemotherapy-induced EMT marker changes (restoring E-cadherin expression, reducing ZEB-1 and CD90) and decreased the CD133^+^CXCR4^+^ stem-like resistant cell population (152).

LY2510924 is a cyclic peptide CXCR4 antagonist that has undergone extensive preclinical and clinical evaluation. Preclinically, LY2510924 specifically blocks CXCL12 binding to CXCR4 with an IC50 of 0.079 nmol/L and inhibits CXCL12-induced tumor cell migration with an IC50 of 0.26 nmol/L (153). In orthotopic CRC xenograft models, LY2510924 combined with 5-fluorouracil significantly suppressed primary tumor growth and reduced metastasis, although the efficacy varied across CRC cell lines depending on the abundance of tumor-initiating cells. A phase I clinical trial evaluated LY2510924 in combination with the PD-L1 inhibitor durvalumab in patients with advanced refractory solid tumors, including CRC. The study established 40 mg daily as the recommended phase II dose, with a favorable safety profile and 44% of patients achieving stable disease as the best response (154).

Other CXCR4 antagonists have also been developed. MSX-122 exhibits strong binding affinity for the extracellular loop 2 (ECL2) of CXCR4 (155). High-affinity, long-acting antagonists such as motixafortide (BKT140) and mavorixafor have attracted considerable attention; these agents are primarily used for hematopoietic stem cell mobilization in patients undergoing autologous stem cell transplantation (156, 157).

Beyond direct anti-tumor effects, CXCR4 antagonism holds significant promise for enhancing immunotherapy. Activation of the CXCL12/CXCR4 axis not only promotes chemotaxis of immunosuppressive cells within the tumor microenvironment but also impairs normal immune cell trafficking, contributing to tumor immune escape (49, 158). In a phase I clinical study involving microsatellite stable (MSS) CRC patients who had failed prior PD-1 immunotherapy, plerixafor treatment significantly increased intratumoral T-cell infiltration and induced expression of multiple immune genes associated with CD8^+^T cell cytotoxic function, thereby enhancing antitumor immunity (49).

Combination strategies have demonstrated efficacy in preclinical CRC models. In the AOM/DSS-induced colitis-associated CRC mouse model, CXCR4 overexpression significantly increased tumor burden, whereas plerixafor treatment markedly reduced tumor development, alleviated intestinal inflammation, and partially restored normal colonic architecture (61). In transplantable CT26 and SL4 mouse models, CXCR4 blockade reduced recruitment of immunosuppressive monocytes and neutrophils, and when combined with anti-angiogenic therapy, further improved therapeutic efficacy (97). Clinical studies have corroborated these findings: continuous infusion of AMD3100 in patients with metastatic pancreatic and colorectal cancers induced a coordinated immune response within tumor tissue and increased expression of immune genes associated with CD8^+^ T cell cytotoxic activity (49).

The immunomodulatory functions of the CXCL12/CXCR4 axis are particularly relevant in the context of immune checkpoint blockade (ICB). MSS colorectal cancer, which constitutes approximately 85% of metastatic CRC, is typically unresponsive to PD-1/PD-L1 inhibitors due to its “cold” immune phenotype characterized by low T cell infiltration (159–162). Preclinical and early clinical studies suggest that CXCR4 blockade can increase intratumoral CD8^+^ T cell infiltration and improve ICB efficacy. In MSS CRC patients who had failed prior therapy, continuous one-week infusion of plerixafor significantly increased intratumoral T cell infiltration and induced an “integrated immune response” signature resembling that observed in ICB-responsive tumors (49). Accordingly, combining CXCR4 inhibitors with immune checkpoint inhibitors (ICI) may provide a promising strategy to overcome immune resistance and convert immunologically “cold” MSS CRC into inflamed, treatment-responsive tumors. These findings support ongoing clinical trials evaluating CXCR4 inhibitor-ICB combinations in gastrointestinal malignancies, including CRC.

Despite strong preclinical evidence supporting the therapeutic potential of CXCL12/CXCR4 axis targeting in CRC, its clinical translation has been limited due to key challenges, including signaling redundancy—where inhibiting CXCR4 alone may upregulate alternative receptors to sustain downstream pathways and complicate CXCL12 regulation—and spatial heterogeneity of CXCL12 expression in the tumor microenvironment, which causes uneven ligand distribution and poor drug penetration (163, 164). Additionally, the axis’s context-dependent dual immune roles make indiscriminate CXCR4 inhibition risky, CXCR4 antagonist monotherapy shows modest effects given CRC’s multifactorial progression, and the lack of standardized predictive biomarkers hinders patient stratification. Collectively, these limitations highlight the CXCL12/CXCR4 network’s complexity, emphasizing the need for future strategies to shift from single-agent targeting to rational combinations, improved biomarker-guided selection, and deeper understanding of context-specific signaling.

## 5 Conclusion and outlook

### 5.1 Conclusion

The CXCL12/CXCR4 axis serves as a central regulatory hub in CRC metastasis, driving tumor progression through multifaceted mechanisms. Through dynamic crosstalk with the PI3K/Akt, Wnt/β-catenin, and Ras/ERK pathways, this axis coordinately regulates the following key processes: (1) driving migration and invasion :acting as a master chemoattractant, the axis directs CXCR4-expressing tumor cells toward CXCL12-rich organs, establishing site-specific metastasis. It further promotes epithelial–mesenchymal transition, ECM degradation via MMP-2/9, and integrin-mediated adhesion, enabling tumor cells to breach tissue barriers; (2) Angiogenesis and lymphangiogenesis: synergizing with VEGF/VEGFR and regulated by hypoxia-induced HIF-1α stabilization; and (3) Shaping the tumor immune microenvironment: establishing an immunosuppressive niche in which differential CXCR4 expression favors the recruitment of immunosuppressive populations (Tregs, M2 macrophages, MDSCs) while excluding effector T cells via CXCR4 internalization and CXCL12–KRT19 physical barriers. Preclinical studies have demonstrated that CXCR4 antagonists (e.g., plerixafor, Peptide R, LY2510924) effectively suppress metastasis and, when combined with immune checkpoint inhibitors, can convert the immunologically “cold” phenotype of microsatellite−stable CRC into an inflamed, treatment−responsive state. Despite these promising results, clinical translation faces challenges including compensatory upregulation of alternative receptors such as CXCR7, spatial heterogeneity of CXCL12 expression, and the lack of validated predictive biomarkers. Thus, the CXCL12/CXCR4 axis represents a highly promising therapeutic target; further elucidation of its regulatory networks and optimization of combination strategies will be essential to achieve durable clinical benefit for patients with metastatic CRC.

### 5.2 Outlook

1. Mechanistic clarity: The potential pathway by which CXCL12/CXCR4 activates Wnt/β-catenin signaling through PI3K/Akt-mediated GSK3β inhibition requires further validation using gene-editing models such as CRISPR. Additionally, the specific regulatory mechanisms of CXCR4 heterodimerization with receptors such as CXCR7 and CCR2 in CRC urgently need to be elucidated.
2. Therapeutic optimization:Dual-target combination strategies (e.g., CXCR4 antagonists combined with PD-1/PD-L1 inhibitors) should be prioritized to overcome compensatory pathway activation and microenvironment-mediated resistance. Concurrently, synergistic regimens combining CXCR4 antagonists with chemotherapy and anti-angiogenic therapy warrant further exploration.
3. Clinical translation: Accelerate phase II/III clinical trials of CXCR4 inhibitors in metastatic CRC cohorts, particularly evaluating their value in reversing immune resistance in MSS-type “cold” tumors. Develop patient stratification strategies based on CXCL12/CXCR4/CXCR7 expression profiles to enable precision therapy.
4. Biomarker Development: Further validate the clinical value of combined CXCR4, CXCR7, and CXCL12 markers in predicting metastasis risk and immunotherapy response, promoting their translation into clinically applicable companion diagnostic tools. In summary, the CXCL12/CXCR4 axis represents a highly promising therapeutic target. In-depth elucidation of its regulatory network and optimization of combination treatment strategies hold the potential to bring clinical breakthroughs for patients with metastatic CRC.

## Glossary

ACKR3: Atypical Chemokine Receptor 3
Akt: protein kinase B
AOM: Azoxymethane
APC: Antigen-Presenting Cell
CAF: Cancer-Associated Fibroblast
CCR2: C-C Chemokine Receptor Type 2
ceRNA: Competing Endogenous RNA
CK1: Casein Kinase 1
CLG: Chanling Gao
CMS: Consensus Molecular Subtype
CRC: colorectal cancer
CXCL12: C-X-C motif chemokine 12
CRLM: Colorectal Cancer Liver Metastasis
Dnmt: DNA Methyltransferase
DPPIV: Dipeptidyl Peptidase IV
DSS: Dextran Sulfate Sodium
Dvl: Dishevelled
ECL2: Extracellular Loop 2
ECM: Extracellular Matrix
EMT: Epithelial-Mesenchymal Transition
ERK: Extracellular Signal-Regulated Kinase
FAK: Focal Adhesion Kinase
FAP: Familial Adenomatous Polyposis
FOXO: Forkhead Box O
GPCR: G Protein-Coupled Receptor
G-MDSC: Granulocytic Myeloid-Derived Suppressor Cell
GRK: G Protein-Coupled Receptor Kinase
GSK3β: Glycogen Synthase Kinase 3 Beta
GZMK: Granzyme K
CAF: Cancer-Associated Fibroblas
CCR2: C-C Chemokine Receptor Type 2
CK1: Casein Kinase 1
HSC: Hepatic Stellate Cell
HIF-1α: Hypoxia-inducible factor-1α
HMGB1: High Mobility Group Box 1
HT-29: Human Colon Cancer Cell Line HT-29
HCT116: Human Colon Cancer Cell Line HCT116
HY-4: DNA, Aptamer Targeting Nucleolin
ICB: Immune Checkpoint Blockade
ICI: Immune Checkpoint Inhibitor
IFN-γ: Interferon-Gamma
IL: Interleukin
JNK: c-Jun N-Terminal Kinase
KRAS: Kirsten Rat Sarcoma Viral Oncogene Homolog
LAMA4: Laminin Subunit Alpha 4
LEF: Lymphoid Enhancer-Binding Factor
MAPK: Mitogen-Activated Protein Kinase
MDSC: Myeloid-Derived Suppressor Cell
MEK: Mitogen-activated extracellular kinase
MIF: Macrophage Migration Inhibitory Factor
miRNA/miR: MicroRNA
MMP: Matrix Metalloproteinase
M-MDSC: Monocytic Myeloid-Derived Suppressor Cell
MSC: Mesenchymal Stem Cell
MSS: Microsatellite Stable
mTOR: Mammalian Target of Rapamycin
NCL: Nucleolin
NF-κB: Nuclear Factor Kappa B
NK cell: Natural Killer Cell
PD-L1: Programmed Death-Ligand 1
PDK1: Phosphoinositide-Dependent Kinase 1
PI3K: Phosphatidylinositol 3-kinase
PIP2: Phosphatidylinositol 4,5-Bisphosphate
PIP3: Phosphatidylinositol 3,4,5-Trisphosphate
PRL-3: Phosphatase of Regenerating Liver-3
PTEN: phosphatase and tensin homolog
Ras: rat sarcoma
RhoA: Ras Homolog Family Member A
ROCK: Rho-Associated Protein Kinase
RTK: Receptor Tyrosine Kinase
SDF-1: Stromal Cell-Derived Factor 1
SFK: SRC Family Kinase
SPOCD1: SPOC Domain-Containing Protein 1
STAT: Signal Transducer and Activator of Transcription
SW480/SW620: Human Colon Cancer Cell Lines
TGFBRII: Transforming Growth Factor Beta Receptor II
TGF-β: Transforming Growth Factor-β
Treg: Regulatory T Cell
TME: Tumor Microenvironment
TNF-α: Tumor Necrosis Factor Alpha
TRPC5: Transient Receptor Potential Cation Channel Subfamily C Member 5
TP53: Tumor Protein 53
THBS1: Thrombospondin 1
TGM2: Transglutaminase 2
VEGF: Vascular Endothelial Growth Factor
VEGFR: Vascular Endothelial Growth Factor Receptor.
